# Effect of Different Cultivation Modes (Photoautotrophic, Mixotrophic, and Heterotrophic) on the Growth of *Chlorella* sp. and Biocompositions

**DOI:** 10.3389/fbioe.2021.774143

**Published:** 2021-12-17

**Authors:** Hyun-Sik Yun, Young-Saeng Kim, Ho-Sung Yoon

**Affiliations:** ^1^ School of Life Sciences, BK21 FOUR KNU Creative BioResearch Group, Kyungpook National University, Daegu, South Korea; ^2^ Research Institute of Ulleung-do & Dok-do, Kyungpook National University, Daegu, South Korea; ^3^ Advanced Bio-Resource Research Center, Kyungpook National University, Daegu, South Korea; ^4^ Department of Biology, College of Natural Sciences, Kyungpook National University, Daegu, South Korea

**Keywords:** biomass yield, chlorella, heterotrophic, mixotrophic, photoautotrophic

## Abstract

In the past, biomass production using microalgae culture was dependent on inorganic carbon sources as microalgae are photosynthetic organisms. However, microalgae utilize both organic and inorganic carbon sources, such as glucose. Glucose is an excellent source of organic carbon that enhances biomass yield and the content of useful substances in microalgae. In this study, photoautotrophic, mixotrophic, and heterotrophic cultivation conditions were applied to three well-known strains of *Chlorella* (KNUA104, KNUA114, and KNUA122) to assess biomass productivity, and compositional changes (lipid, protein, and pigment) were evaluated in BG11 media under photoautotrophic, mixotrophic, and heterotrophic conditions utilizing different initial concentrations of glucose (5, 10, 15, 20, and 25 g L^−1^). Compared to the photoautotrophic condition (biomass yield: KNUA104, 0.35 ± 0.04 g/L/d; KNUA114, 0.40 ± 0.08 g/L/d; KNUA122, 0.38 ± 0.05 g/L/d) glucose was absent, and the biomass yield improved in the mixotrophic (glucose: 20 g L^−1^; biomass yield: KNUA104, 2.99 ± 0.10 g/L/d; KNUA114, 5.18 ± 0.81 g/L/d; KNUA122, 5.07 ± 0.22 g/L/d) and heterotrophic conditions (glucose: 20 g L^−1^; biomass yield: KNUA104, 1.72 ± 0.26 g/L/d; KNUA114, 4.26 ± 0.27 g/L/d; KNUA122, 4.32 ± 0.32 g/L/d). All strains under mixotrophic and heterotrophic conditions were optimally cultured when 15–20 g L^−1^ initial glucose was provided. Although bioresourse productivity improved under both mixotrophic and heterotrophic conditions where mixotrophic conditions were found to be optimal as the yields of lipid and pigment were also enhanced. Protein content was less affected by the presence of light or the concentration of glucose. Under mixotrophic conditions, the highest lipid content (glucose: 15 g L^−1^; lipid content: 68.80 ± 0.54%) was obtained with *Chlorella vulgaris* KNUA104, and enhanced pigment productivity of *Chlorella sorokiniana* KNUA114 and KNUA122 (additional pigment yield obtained with 15 g L^−1^ glucose: KNUA 114, 0.33 ± 0.01 g L^−1^; KNUA122, 0.21 ± 0.01 g L^−1^). Also, saturated fatty acid (SFA) content was enhanced in all strains (SFA: KNUA104, 29.76 ± 1.31%; KNUA114, 37.01 ± 0.98%; KNUA122, 33.37 ± 0.17%) under mixotrophic conditions. These results suggest that mixotrophic cultivation of *Chlorella vulgaris* and *Chlorella sorokiniana* could improve biomass yield and the raw material quality of biomass.

## Introduction

Microalgae survive through photosynthesis (Masojıdek et al., 2004), and rely on light energy to perform biosynthesis using both trace elements and major elements including carbon, nitrogen, and phosphorus (Zhu et al., 2021a; Zhu et al., 2021b; Yaakob et al., 2021). Such growth conditions are called photoautotrophic. The biomass productivity of microalgae using photosynthesis is higher compared to terrestrial plants producing useful materials such as fatty acids and pigments (Brennan and Owende, 2010; Singh et al., 2011; Ma et al., 2020; Yaakob et al., 2021). For industrial applications of microalgae, methods to produce large amounts of biomass are required (Priyadarshani and Rath, 2012). Large-scale open ponds and photobioreactors systems based on photosynthesis have been used as such methods (Ugwu et al., 2008; Kumar et al., 2015; Zhu et al., 2019a; Zhu et al., 2019b). However, there are limitations with the use of cultures based on photosynthesis (Zhu et al., 2019a; Zhu et al., 2019b). Firstly, the transmittance of light, the sole energy source, decreases as the cell density increases (Zhu et al., 2019a; Zhu et al., 2019b) making this a limiting factor. Secondly, the content of useful substances in the produced biomass is low (Merzlyak et al., 2007). Although nutrient starvation and osmotic stress have been suggested as strategies to enhance the production of useful substances (Merzlyak et al., 2007), such stresses can reduce overall biomass productivity without increasing the content of target substances (Chen et al., 2017).

Although microalgae can utilize inorganic carbon sources for photosynthesis (Zhu et al., 2021a; Zhu et al., 2021b), the biomass productivity of these microalgae is low and limited (Verma et al., 2020). To maximize the production of high-quality biomass, it has been proposed to use a culture method in which an organic carbon source is provided to microalgae (Roostaei et al., 2018). By providing an organic carbon source, biomass productivity is improved by increasing growth efficiency and cell density (Roostaei et al., 2018). In addition, the content of useful substances, including lipids and pigment, can be enhanced (Ip et al., 2004; Liang et al., 2009). The application of the organic carbon source can be divided into two types depending on the presence (mixotrophic) or absence (heterotrophic) of light (Liang et al., 2009; Bassi et al., 2014). Mixotrophic cultivation is preferred due to several drawbacks with heterotrophic cultivation (Liu et al., 2009; Perez-Garcia et al., 2011; Wang et al., 2014). When cultivating microalgae under heterotrophic conditions, the light-blocked environment makes photosynthesis impossible (Chojnacka and Marquez-Rocha, 2004), meaning the only source of energy is organic carbon, which causes CO_2_ to be released (Chojnacka and Marquez-Rocha, 2004). Under heterotrophic conditions, the released CO_2_ cannot be used as a carbon source for photosynthesis, leading to the lowering of the pH of the culture by the emitted CO_2_ (Chojnacka and Marquez-Rocha, 2004). This change in pH affects the growth rate of microalgae, which directly affects biomass productivity (Moheimani, 2013; Bartley et al., 2014). By contrast, mixotrophic cultivation avoids these drawbacks of heterotrophic cultivation (Lowrey et al., 2015), as both organic carbon and light energy are available (Cheirsilp and Torpee, 2012; Lowrey et al., 2015). Therefore, it is possible to culture microalgae to high densities, leading to enhanced biomass productivity (Chen and Zhang, 1997; Kong et al., 2013). Although heterotrophic and mixotrophic conditions both improve biomass productivity compared to photoautotrophic conditions, mixotrophic cultivation is preferred as it has greater biomass productivity (Liang et al., 2009; Bassi et al., 2014; Lowrey et al., 2015).

In previous studies, two cultivation methods that provided organic carbon sources were applied to various microalgae (Bassi et al., 2014; Engin et al., 2018). It was shown that this method had economically efficient culture conditions for microalgae in a process that was aimed toward the production of bioresource and fatty acid for bioenergy development (Bassi et al., 2014; Engin et al., 2018). Currently, microalgae are utilized for bioenergy, food supplement, and medicinal compound production (López et al., 2019; Menegol et al., 2019). By applying mixotrophic and heterotrophic conditions, the accumulation of useful substances and biomass productivity in microalgae has been evaluated (López et al., 2019; Menegol et al., 2019). *Chlorella*, a microalgae genus that is useful for industrial applications, has also been used in research and development (Priyadarshani and Rath, 2012; Safi et al., 2014). For example, *Chlorella zofingiensis* and *Chlorella vulgaris* have been studied as sources of unsaturated fatty acids and astaxanthin (Ip et al., 2004; Kumar et al., 2019). In addition, *Chlorella sorokiniana*, which produces large amounts of carotenoid pigments (including lutein), is being evaluated for its industrial uses as a microalgae species (Chen et al., 2016).

Previous studies have determined the optimal growth temperature conditions for *Chlorella vulgaris* and *Chlorella sorokiniana* (Yun et al., 2020). Furthermore, it was found that an organic carbon source in a heterotrophic condition could be utilized by *Chlorella* (Kim et al., 2020). In this study, we evaluated the applicability and usability of glucose as an organic carbon source for *Chlorella vulgaris* (KNUA104) and *Chlorella sorokiniana* (KNUA114 and KNUA122) strains under heterotrophic and mixotrophic conditions. Glucose consumption, biomass productivity, and content of target substances were measured and analyzed for each strain. Based on these results, the optimal culture conditions for *Chlorella vulgaris* and *Chlorella sorokiniana* strains were identified and the value of produced substances as biomaterials was demonstrated under heterotrophic and mixotrophic conditions.

## Materials and Methods

### Microalgal Strains and Cell Preparation

The unicellular green algae strain *Chlorella sorokiniana* KNUA114, KNUA122, and *Chlorella vulgaris* KNUA104 isolated from Ulleung Island were utilized in this study. The *Chlorella* strains were maintained at 25°C on agar plates of BG11 medium consisting of the following (per liter): 1,500 mg NaNO_3_, 36 mg CaCl_2_ 2H_2_O, 12 mg ferric ammonium citrate, 1.1 mg EDTA Na_2_ 2H_2_O, 40 mg K_2_HPO_4_, 75 mg MgSO_4_ 7H_2_O, 20 mg Na_2_CO_3_, 2.86 mg H_3_BO_3_, 1.81 mg MnCl_2_ 4H_2_O, 0.222 mg ZnSO_4_ 7H_2_O, 0.39 mg Na_2_MoO_4_ 2H_2_O, 0.079 mg CuSO_2_ 5H_2_O, and 0.049 mg Co(NO_3_)_2_ 6H_2_O (Yang et al., 2015). For the experiments, the *Chlorella* strains were cultured in a modified BG11 medium for 10 days at 25°C in an incubation room on an orbital shaker at 160 rpm. The cultured cells were collected by centrifugation at 4,000 rpm for 10 min and washed twice with sterile distilled water. The washed cells were then resuspended in fresh BG11 medium (resuspended cell density: OD_680_ = 0.30 ± 0.02; cell density: KNUA104, 223.67 ± 16.81 × 10^4^ cells ml^−1^; KNUA114, 877.00 ± 39.74 × 10^4^ cells ml^−1^; KNUA122, 718.33 ± 3.06 × 10^4^ cells ml^−1^) and stored at 4°C. The cultured cells collected were used as biological replicates in three repeated experiments in this study.

### Photoautotrophic, Mixotrophic and Heterotrophic Cultivation

For photoautotrophic cultivation pure BG11 medium was used, and for the cultivation of mixotrophic and heterotrophic, glucose was added as an organic carbon source. Glucose was sterilized and supplemented at several concentrations (5, 10, 15, 20, and 25 g L^−1^). Each *Chlorella* strain (diluted to approximately 0.3 at OD_680_, 15 ml) was inoculated into 150 ml of medium in a 250 ml flask. Two incubation rooms were used to apply the required conditions for each cultivation as follows: the photoautotrophic and mixotrophic cultures were incubated in an illuminated incubation room (light source, fluorescent lamp; photosynthetic photon flux density, approximately 55 μmol m^−2^ s^−1^; light: dark cycle, 16:8 h). To block light effect, heterotrophic cultures were incubated in an unilluminated incubation room and the flasks were covered with aluminum foil. All culture flasks were incubated at 25°C in an orbital shaker rotating at 160 rpm.

### Growth Analysis

The growth of each strain was assessed as dry weight (Yoo et al., 2010). To determine the dry weight, a 5 ml aliquot of each culture was filtered through a pre-weighed glass fiber filter. The cell pellet was washed with distilled water, oven-dried 70°C of for 12 h, and then weighed (Yoo et al., 2010; Kim et al., 2017). Cell morphology and the culture media were assessed every 2 days. For the cell morphology assessments, 1 ml of culture was centrifuged at 4,000 rpm for 5 min. The cell pellet was subsequently washed with distilled water and inspected using a biological Microscope. Glucose consumption during growth was calculated by measuring the concentration of glucose remaining in the culture medium using the anthranone method (Chu et al., 2018) As anthranone is not specific for glucose, an electrochemical glucose meter (Daeilpharm, Seongnam, South Korea) was used to exclude the measurement of other sugars (Wang, 2008).

### Biomass Collection and Chemical Analysis

Samples were harvested when cells were either in the log phase (4 days in culture) or stationary phase (10 days in culture). Cells were collected by centrifugation at 4,000 rpm for 20 min, and subsequently freeze-dried. Samples of 10 mg were weighed and placed into tubes. Total lipid content was determined by the sulpho-phospho-vanillin (SPV) colorimetric method (Mishra et al., 2014) using canola oil to generate the standard curve and equations. Samples were suspended in 1 ml distilled water and sonicated for 10 s. Next, the sonicated samples were processed according to the SPV method, and the OD_530_ of the supernatant was measured (Mishra et al., 2014). Total protein content was determined using the Bradford method (Bradford, 1976), using bovine serum albumin as a standard to generate the standard curve and equations. Samples were suspended in 1 ml phosphate buffer and sonicated (Meijer and Wijffels, 1998). Proteins were extracted, and the protein concentration was calculated from the OD_562_ as previously published (Bradford, 1976). For the measurement of pigment contents, samples were sonicated. Methanol was used to extract the pigment (Şükran et al., 1998). The OD_666_, OD_653_, and OD_470_ values of the extracted pigments were measured (de Souza et al., 2019; Niroula et al., 2019) and pigment content was calculated according to the formulas of Lichtentaler and Wellburn (de Souza et al., 2019; Niroula et al., 2019).

A mixture of chloroform: methanol (1:1) was used to extract lipids from 30 mg of freeze-dried and pulverized microalgae to analyze fatty acid composition (Yeo et al., 2011). Chloroform was removed from the extracted mixture using an evaporator, and the extracted lipid was then treated with a solution of methanol and potassium solution to facilitate transesterification. To isolate fatty acid methyl esters (FAME), hexane was added to the extracted lipid mixtureand then stirred at 30°C for 10 h. The hexane layer was isolated from the mixture and analyzed by gas chromatography (SUPELCO, Bellefonte, PA, United States of America) as an external standard to characterize the composition of FAME. Gas chromatography was performed with a 6890N gas chromatograph (Agilent Technology Inc., Santa Clara, CA) equipped with a 5973N mass selective detector (Agilent Technologies) and an HP-5MS capillary column [30 m × 0.25 mm (internal diameter) × 0.25 μm film thickness; Agilent Technologies] (Furuhashi and Weckwerth, 2013).

### Statistical Analysis

The ratio of total lipid contents and the total protein contents was defined as 100%. Individual data points were compared using a Student’s *t*-test, and a *p*-value of <0.05 was considered statistically significant. All experiments were performed at least in triplicate, and the general microbiology test data were expressed as mean ± standard deviation (SD) (*n* = 3).

## Results and Discussion

### Effects of Culture Conditions on Microalgal and Cell Morphology

**FIGURE 1:**
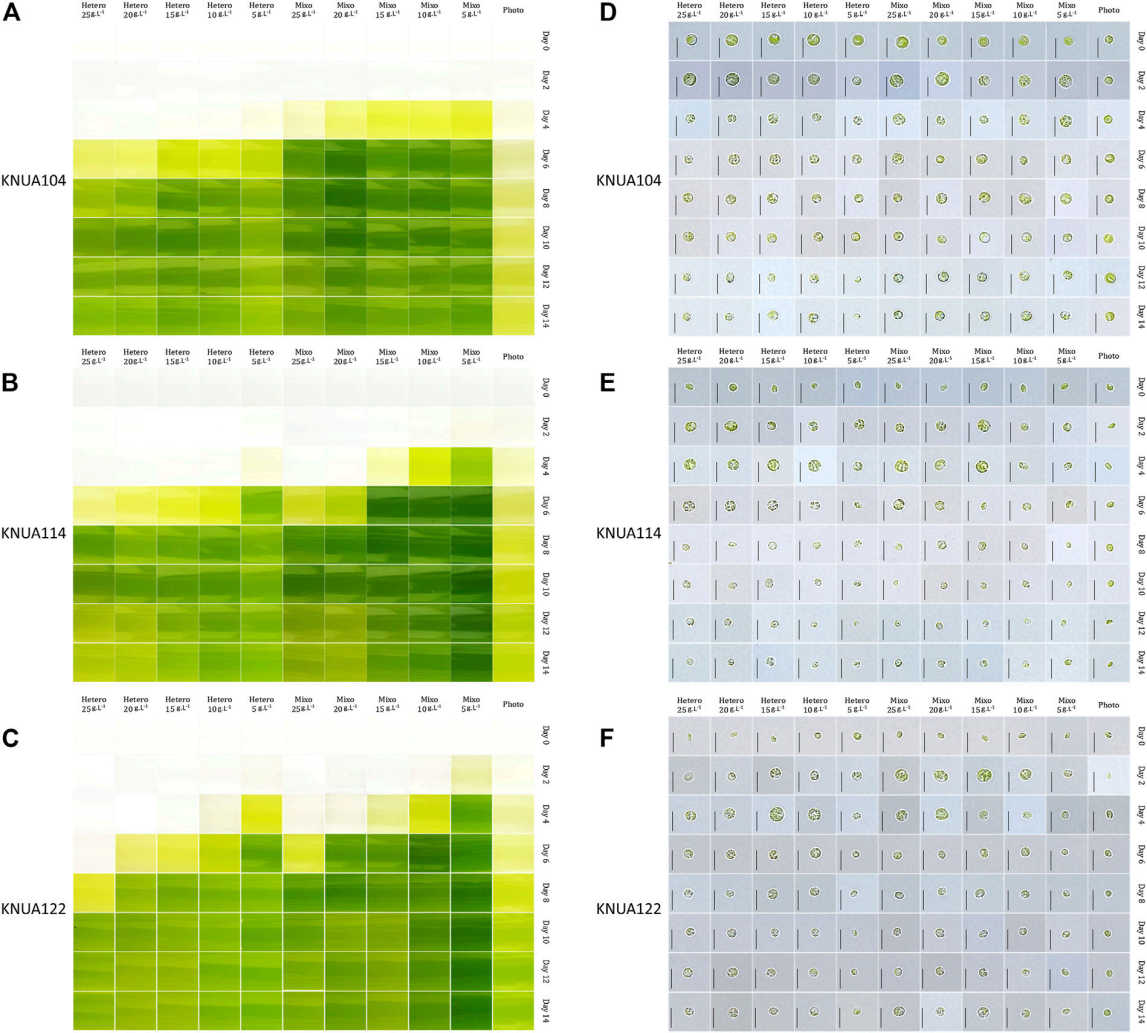
Color of the algal culture media at the indicated times for KNUA104 **(A)**, KNUA114 **(B)**, and KNUA122 **(C)** strains. Light microscopic images of KNUA104 **(D)**, KNUA114 **(E)**, and KNUA122 **(F)** strains. The culture conditions for heterotrophic (Hetero), mixotrophic (Mixo), and photoautotrophic (Photo). The initial glucose concentrations are displayed at the top of each set of panels, and the incubation times are indicated along the left of each panel. Scale bar = 5 µm.

The *Chlorella* strains cultured with various initial glucose concentrations under mixotrophic and heterotrophic conditions were periodically sampled during culture. The color of the culture and the morphology characteristics of the *Chlorella* strains are detailed in Figure 1. The OD_680_ is used to measure the growth of microalgae, including *Chlorella*, in many previous studies (Leng et al., 2020; Vu et al., 2020). Herethe OD_680_ of three strains at stationary phase was lower than 2.00, the maximum OD_680_ value under photoautotrophic conditions (optical density at 680 nm: KNUA104, 1.42 ± 0.01; KNUA114, 1.77 ± 0.02; KNUA122, 1.74 ± 0.02). However, 4–6 days after the start of cultivation, for the mixotrophic and heterotrophic conditions the OD_680_ value exceeded the measurement limit of optical density (maximum measurable value of spectrophotometer: 3.00, Mecasys, South Korea) (Swinehart, 1962). As an alternative to measuring cell density by OD_680_ value, the color of the culture was recorded for each sample. There was a change in the microalgae culture for each cultivation condition we applied, and this also occurred in the pigment (Leng et al., 2020; Vu et al., 2020). During log-phase growth, the color of the culture for samples grown under mixotrophic conditions is generally more intense compared to samples grown under heterotrophic conditions the color of the culture varying according to the initial glucose concentration. In the early stages (0–4 days), the color of the culture is more intense at lower initial glucose concentrations. However, in later stages (10–14 days), the color of the culture medium changes from green to light green, or yellow at higher initial glucose concentrations. The morphology and cell size of the chloroplast had several variations during cultivation, as shown in Figure 1 (Azaman et al., 2017). Cup-shaped chloroplasts, commonly observed in strains of the *Chlorella* genus (Safi et al., 2014), are not seen in these experiments; instead, small dot-shaped granules with an uneven shape are distributed throughout the cell. Starting on the second day of incubation, cells grown under mixotrophic and heterotrophic conditions are larger in comparison to those grown under photoautotrophic conditions. Also, the shape of KNUA114 and KNUA122 cells changed from elliptical to circular. With an increase in culture time, the amount of green space (occupied by chloroplasts) in the cells decreased, and the color of the chloroplasts changed from dark green to light green. These trends are particularly evident in *Chlorella sorokiniana* KNUA114 and KNUA122, suggesting that high-density cultivation of *Chlorella* was obtained by applying mixotrophic or heterotrophic conditions. In addition, the variations in chloroplasts under mixotrophic and heterotrophic conditions indicate that the presence of glucose in the medium causes variations not only in cell density but also in the chloroplasts in the cells.

### Effects of Glucose Concentration on Growth

**FIGURE 2:**
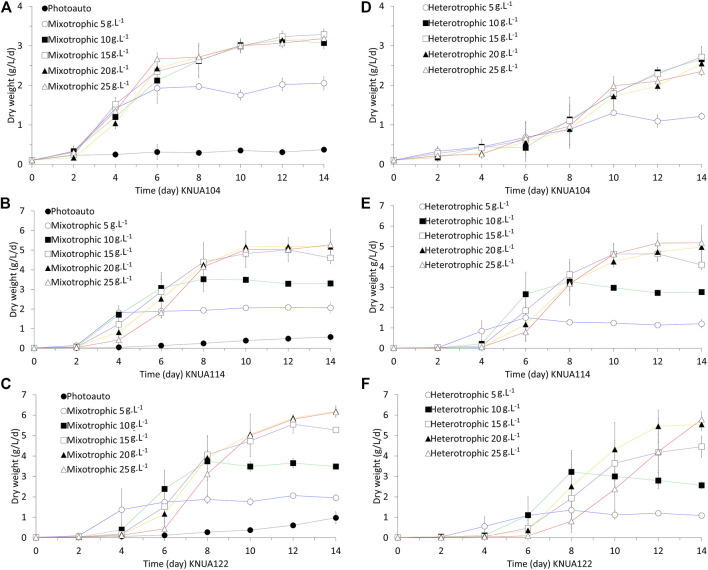
Growth of *Chlorella* under photoautotrophic (Photoauto), mixotrophic, and heterotrophic conditions as measured by dry weight. KNUA104 **(A)**, KNUA114 **(B)**, and KNUA122 **(C)** under photoautotrophic and mixotrophic conditions. KNUA104 **(D)**, KNUA114 **(E)**, and KNUA122 **(F)** under heterotrophic conditions. Marked characteristics are annotated in Table 1.

To determine the effect of culture conditions on the growth of *Chlorella,* the dry weight was measured. The measured results used to confirm the growth patterns observed qualitatively by color and morphology are shown in Figure 2. The effects of glucose on the growth of *Chlorella* under mixotrophic and heterotrophic conditions (at various concentrations of glucose) were investigated. Among all conditions, the growth of *Chlorella* under photoautotrophic conditions was the slowest (Figure 2). The maximum dry weight measured in photoautotrophic conditions was measured 10–12 days under photoautotrophic conditions (KNUA104: 0.35 ± 0.04 g L^−1^; KNUA114: 0.40 ± 0.08 g L^−1^; KNUA122: 0.38 ± 0.05 g L^−1^), whereas higher dry weights were measured 4–6 days under mixotrophic and heterotrophic conditions. Furthermore, during any point in the cultivation period, all mixotrophic and heterotrophic conditions had higher dry weights than those measured under photoautotrophic conditions. For *Chlorella vulgaris* KNUA104 (Figures 2A,D), the effect of initial glucose concentration on dry weight is similar to glucose concentrations above 5 g L^−1^ under mixotrophic and heterotrophic conditions. Moreover, the maximum dry weight is observed at 10–12 days under mixotrophic conditions and at 12–14 days under heterotrophic conditions. The highest dry weights were observed for *Chlorella vulgaris* KNUA1 at glucose concentrations above 10 g L^−1^ (mixotrophic conditions: 3.02 ± 0.10 g L^−1^; heterotrophic conditions: 1.78 ± 0.45 g L^−1^). In the case of *Chlorella sorokiniana* KNUA114 (Figures 2B,E) and KNUA122 (Figures 2C,F), the growth rate in the early stages of cultivation is lower with a higher glucose concentration. Furthermore, the maximum dry weight differs for each condition. KNUA114 strains reach stationary phase approximately 1–2 days earlier than KNUA122 strains (KNUA114, mixotrophic conditions: 5 g L^−1^, day 4; 10 g L^−1^, day 6; above 15 g L^−1^, day 10; heterotrophic conditions: 5 g L^−1^, day 6; 10 g L^−1^, day 8; above 15 g L^−1^, day 10–12; KNUA122, mixotrophic conditions: 5 g L^−1^, day 4–6; 10 g L^−1^, day 8; above 15 g L^−1^, day 10–12; heterotrophic conditions: 5 g L^−1^ day 6, 10 g L^−1^ day 8, above 15 g L^−1^ day 10–12). The highest dry weight of the tested conditions occurred at glucose concentrations of 15, 20, and 25 g L^−1^ in both KNUA114 (mixotrophic conditions: 4.83 ± 0.20 g L^−1^; heterotrophic conditions: 4.63 ± 0.52 g L^−1^) and KNUA122 (mixotrophic conditions: 4.73 ± 0.19 g L^−1^; heterotrophic conditions: 3.64 ± 0.32 g L^−1^).

Through the analysis of growth patterns by several independent measurements, it was observed that dry weight per unit volume increases through the application of mixotrophic and heterotrophic conditions to KNUA strains (Kong et al., 2013; Li et al., 2014). The dry weight in 14-days KNUA114 and KNUA122 cultures increased proportionally to the concentration of glucose between 5 and 20 g L^−1^ (Figures 2B,C,E,F). However, for each strain, there is a glucose concentration above which there was no notable increase in dry weight (KNUA104, 10 g L^−1^; KNUA114, 15–20 g L^−1^; KNUA122, 15–20 g L^−1^). Of interest is the observation that the rate of dry weight gain in the KNUA114 and KNUA122 strains was higher in the early stages of culture. In addition, the time for these strains to reach the stationary phase was shorter at a lower glucose concentration. Although differing depending on the concentration of glucose, the growth of *Chlorella* was generally promoted in the mixotrophic and heterotrophic conditions (Nouri et al., 2021). Therefore, the growth of *Chlorella* was indeed promoted by glucose (Nouri et al., 2021). Moreover, considering *Chlorella* can not only grow in heterotrophic conditions where photosynthesis is impossible, the growth rate was higher compared to photoautotrophic conditions, demonstrating that glucose as an energy source can replace photosynthesis and promote the growth of *Chlorella* (Shen et al., 2020; Smith et al., 2021). This also applies to the mixotrophic condition and provides an understanding of the superior growth pattern in this condition, which not only allows photosynthesis but also receives the influence of glucose (Shen et al., 2020; Nouri et al., 2021; Smith et al., 2021). These results suggest that mixotrophic and heterotrophic conditions promote the growth of microalgae; however, an optimal glucose concentration for each strain is needed, and strains may experience slower growth at the early stages of cultivation due to osmotic stress.

### Consumption of Glucose and Biomass Yield

**FIGURE 3:**
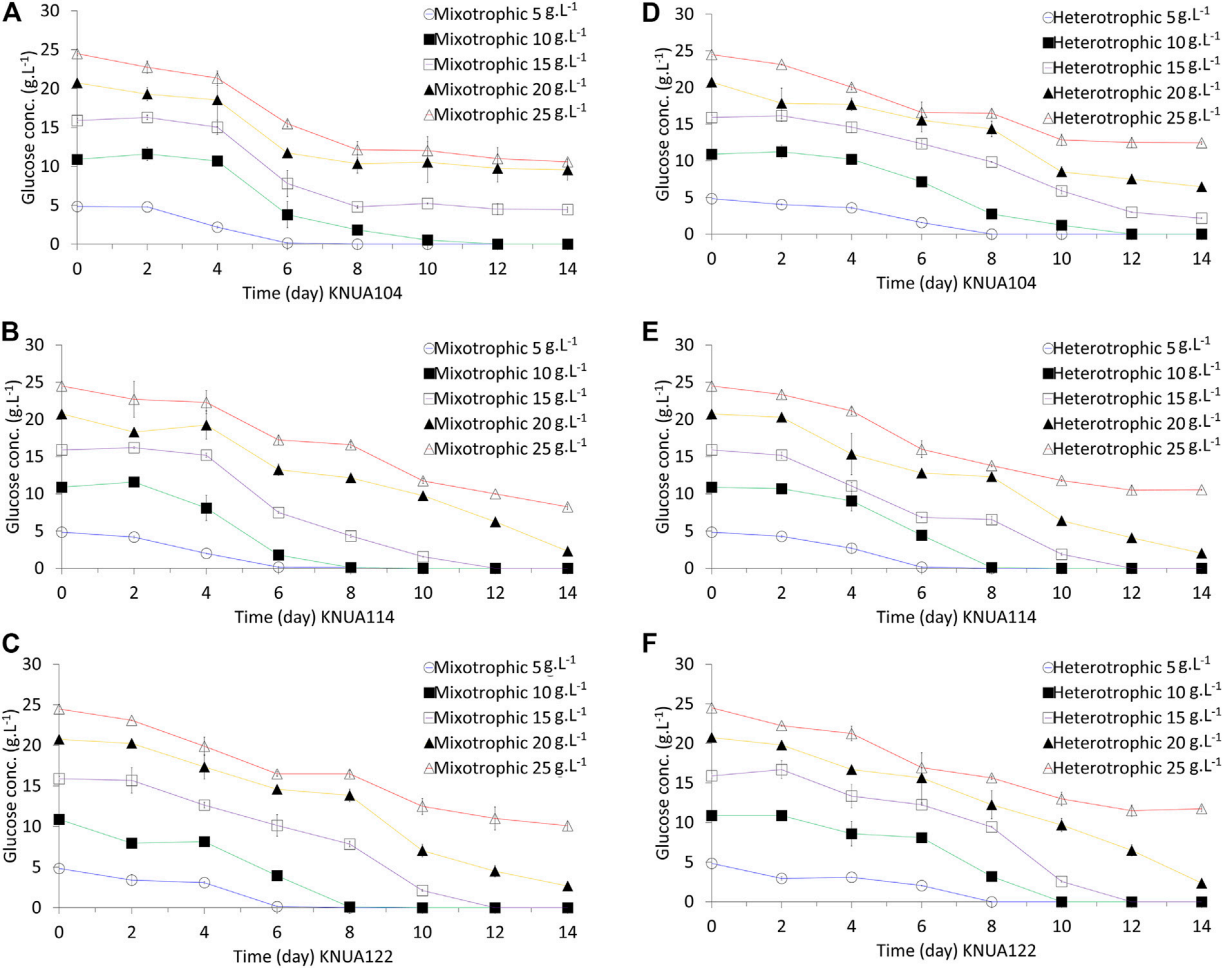
Residual glucose concentrations in cultures of *Chlorella* grown under mixotrophic and heterotrophic conditions. KNUA104 **(A)**, KNUA114 **(B)**, and KNUA122 **(C)** under mixotrophic conditions. KNUA104 **(D)**, KNUA114 **(E)**, and KNUA122 **(F)** grown under heterotrophic conditions. Marked characteristics are annotated in Table 1.

The rate of glucose consumption is an indication of the degree to which strains use glucose as an energy and carbon source during culture (Shi et al., 1999). The residual glucose concentration over time under mixotrophic and heterotrophic conditions has been evaluated in this study (Figure 3). Under each condition, glucose consumption is higher during the log-phase growth (days 4–8) compared to the growth during the lag phase (days 0–2). During the stationary phase (≥day 10) the consumption of glucose reduces. The time required for consuming the same amount of glucose is shorter in mixotrophic cultures than in heterotrophic cultures (Kamiya, 1985). Changes in glucose consumption and the time required to consume a certain amount of glucose are likely to be closely related to the overall growth (Figure 2) (Shi et al., 1999). Table 1 summarizes the characteristics of the biomass productivity observed in these experiments using cultures harvested on day 10 (biomass yield, glucose consumption rate, additional biomass yield, and additional biomass yield per gram of consumed glucose). The highest biomass yield of the three algae strains is at different glucose concentrations does not lead to higher yields (KNUA104, 3.02 ± 0.10 g/L/d; KNUA114, 5.18 ± 0.81 g/L/d; KNUA122, 5.07 ± 0.22 g/L/d). Moreover, the strains exhibit a threshold above which higher initial glucose concentrations (glucose concentration: KNUA104, 10 g L^−1^; KNUA114 and KNUA122, 20 g L^−1^). Additional biomass yield per gram of consumed glucose was calculated using the glucose consumption rate and additional biomass yield. KNUA104 strains exhibited the highest additional biomass yield per gram of consumed glucose (0.29 ± 0.03 g g^−1^, mixotrophic conditions) at an initial glucose concentration of 5 g L^−1^. By contrast, KNUA114 and KNUA122 had the highest additional biomass yield per gram of consumed glucose at an initial glucose concentration of 20 g L^−1^ (KNUA114, 0.47 ± 0.08 g g^−1^, mixotrophic conditions; KNUA122, 0.38 ± 0.03 g g^−1^, heterotrophic conditions). The highest additional biomass yield per gram of consumed glucose was observed in KNUA114 with an initial glucose concentration of 20 g L^−1^ under mixotrophic conditions. Compared with photoautotrophic cultivation, the biomass yield of the three algae strains increased by using mixotrophic and heterotrophic cultivation (Table 1). Mixotrophic conditions increased the productivity by at least 12-fold (KNUA114 and KNUA122) or 8-fold (KNUA104). Whereas heterotrophic conditions increased productivity by at least 9-fold (KNUA114 and KNUA122) or 5-fold (KNUA104). The addition of glucose increases biomass productivity and promotes growth, particularly under mixotrophic conditions that maintain the ability to utilize light energy (Kong et al., 2013). These results suggest that both organic carbon sources and light energy can be used as energy sources, and having two energy sources is, therefore, more efficient (Shi et al., 1999; Kong et al., 2013).

**TABLE 1:** The total biomass yield in *Chlorella* culture under mixotrophic and heterotrophic conditions with varying initial glucose concentrations (zero initial glucose indicates photoautotrophic conditions).

Strain	Initial glucose concentration (g L^−1^)	Biomass yield (g/L/d)	Glucose consumption rate (%, w/w)	Additional biomass yield (g/L/d)	Additional biomass yield per gram of consumed glucose (g g^−1^)
*Chlorella vulgaris* KNUA104	0	0.35 ± 0.04			
	Mixotrophic				
	5	1.76 ± 0.13	98.91 ± 1.01	1.41 ± 0.13	0.29 ± 0.03
	10	3.02 ± 0.10	94.75 ± 7.43	2.67 ± 0.10	0.28 ± 0.01
	15	2.98 ± 0.16	65.00 ± 0.47	2.63 ± 0.16	0.27 ± 0.02
	20	2.99 ± 0.10	47.25 ± 13.08	2.64 ± 0.10	0.28 ± 0.01
	25	3.00 ± 0.18	51.80 ± 7.07	2.65 ± 0.18	0.20 ± 0.01
	Heterotrophic				
	5	1.31 ± 0.08	97.22 ± 1.23	0.96 ± 0.08	0.20 ± 0.02
	10	1.78 ± 0.45	87.25 ± 0.07	1.43 ± 0.45	0.16 ± 0.05
	15	1.79 ± 0.04	60.67 ± 2.82	1.44 ± 0.04	0.16 ± 0.01
	20	1.72 ± 0.26	57.50 ± 3.54	1.37 ± 0.26	0.12 ± 0.02
	25	1.99 ± 0.11	48.60 ± 3.11	1.64 ± 0.11	0.13 ± 0.01
*Chlorella sorokiniana* KNUA114	0	0.40 ± 0.08			
	Mixotrophic				
	5	2.06 ± 0.01	99.13 ± 0.52	1.66 ± 0.01	0.33 ± 0.01
	10	3.49 ± 0.15	99.24 ± 0.41	3.09 ± 0.15	0.31 ± 0.02
	15	4.83 ± 0.20	89.67 ± 0.47	4.43 ± 0.20	0.32 ± 0.01
	20	5.18 ± 0.81	51.25 ± 0.35	4.78 ± 0.81	0.47 ± 0.08
	25	5.04 ± 0.93	53.00 ± 1.41	4.64 ± 0.93	0.35 ± 0.07
	Heterotrophic				
	5	1.24 ± 0.11	98.59 ± 0.18	0.84 ± 0.11	0.17 ± 0.02
	10	2.97 ± 0.06	97.63 ± 0.34	2.57 ± 0.06	0.26 ± 0.01
	15	4.63 ± 0.52	87.43 ± 1.08	4.23 ± 0.52	0.32 ± 0.04
	20	4.26 ± 0.27	68.00 ± 14.14	3.86 ± 0.27	0.28 ± 0.02
	25	4.62 ± 0.50	52.80 ± 0.57	4.22 ± 0.50	0.32 ± 0.04
*Chlorella sorokiniana* KNUA122	0	0.38 ± 0.05			
	Mixotrophic				
	5	1.77 ± 0.19	98.65 ± 0.67	1.39 ± 0.19	0.28 ± 0.01
	10	3.49 ± 0.26	98.58 ± 0.42	3.11 ± 0.26	0.32 ± 0.03
	15	4.73 ± 0.19	85.83 ± 0.70	4.35 ± 0.19	0.34 ± 0.01
	20	5.07 ± 0.22	64.75 ± 3.89	4.69 ± 0.22	0.36 ± 0.02
	25	5.01 ± 1.04	50.00 ± 3.96	4.63 ± 1.04	0.37 ± 0.08
	Heterotrophic				
	5	1.12 ± 0.21	97.43 ± 0.84	0.74 ± 0.21	0.15 ± 0.04
	10	3.00 ± 0.11	98.68 ± 1.23	2.62 ± 0.11	0.27 ± 0.01
	15	3.64 ± 0.32	82.87 ± 0.66	3.26 ± 0.32	0.26 ± 0.03
	20	4.32 ± 0.32	51.50 ± 4.24	3.94 ± 0.32	0.38 ± 0.03
	25	2.38 ± 1.23	48.00 ± 3.39	2.00 ± 1.23	0.17 ± 0.10

### Effects of Culture Conditions on Yields and Content of Lipid, Protein, and Pigment

**FIGURE 4:**
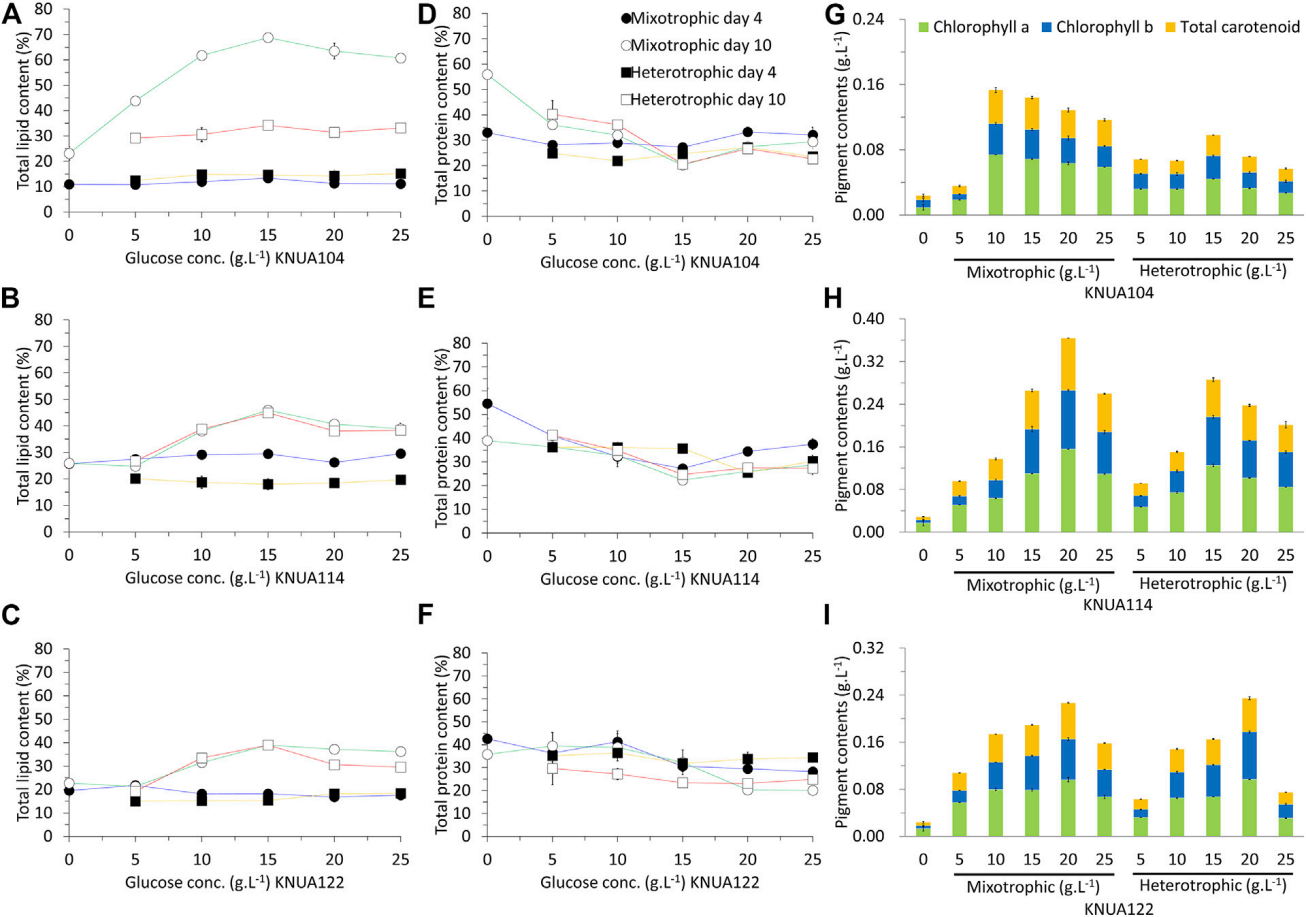
Total lipid content of KNUA104 **(A)**, KNUA114 **(A)**, and KNUA122 **(C)**. Total protein content of KNUA104 **(D)**, KNUA114 **(E)**, and KNUA122 **(F)**. Pigment contents and compositions of KNUA104 **(G)**, KNUA114 **(H)**, and KNUA122 **(I)**. Marked characteristics are annotated in Table 3.

In addition to bioresourse productivity, the content of the biomass produced is important for industrial applications. The content of lipid, protein, and pigment obtained in these experiments is shown in Figure 4. The lipid content of each algae strain was higher in the stationary phase (day 10) compared to the log phase (day 4). Higher initial glucose concentrations were associated with higher lipid content. The highest overall lipid content was observed in *Chlorella vulgaris* KNUA104 at an initial glucose concentration of 15 g L^−1^ under mixotrophic conditions. Mixotrophic cultivation yielded higher lipid content in all three algae strains compared to heterotrophic cultivation. This difference in lipid content between mixotrophic and heterotrophic cultivation of *Chlorella vulgaris* KNUA104 was significant. Moreover, no significant differences were observed in *Chlorella sorokiniana* KNUA114 and KNUA122, regardless of the initial glucose concentration or the presence of light. Although the initial glucose concentration affected the lipid content, the protein content was not significantly affected in all three algae strains. However, the initial glucose concentration affected the pigment content. The highest pigment content for each strain occurred with different initial glucose concentrations (KNUA104: 10 g L^−1^, mixotrophic condition; KNUA114: 20 g L^−1^; mixotrophic condition; KNUA122: 20 g L^−1^, mixotrophic and heterotrophic conditions). Overall, the highest pigment content was produced by KNUA114 under mixotrophic conditions at an initial glucose concentration of 20 g L^−1^. These results demonstrate that the use of mixotrophic and heterotrophic conditions using glucose improves the quality of biomass (Liang et al., 2009; Kong et al., 2013; Li et al., 2014; Chen et al., 2016). Of importance, this improvement does not occur across all components and different conditions cause different effects in each strain (Kong et al., 2013; Chen et al., 2016). The lipid or pigment content in the biomass was improved in the tested strains, although the extent of the improvement differs between strains. These results demonstrate that the strain used must increase the productivity of a specific product. In addition, these results support the application of mixotrophic and heterotrophic conditions to improve the yields of specific components in each strain.

### Effects of Glucose on the Composition of Lipid Contents

The effect that applying mixotrophic cultivation had on the composition of the lipids produced was analyzed, and the results are summarized in Table 2. The fatty acid composition extracted from cultures grown under photoautotrophic and mixotrophic conditions (glucose concentration at 20 g L^−1^) was compared. Under photoautotrophic and mixotrophic conditions, the major fatty acid components of the *Chlorella* strains were C_16:0_, C_18:1_, C_18:2_, and C_18:3_ (above 10%) (Table 2). However, the fatty acid composition of samples differs depending on the cultivation condition and strain, in this experiment the contents of SFA and monounsaturated fatty acid were enhanced under mixotrophic conditions (increase in SFA: approximately 5–10%; increased in monounsaturated fatty acid: approximately 20%). In addition, C_19:0_ was also detected in the mixotrophic conditions and was not detected in the photoautotrophic conditions. In particular, a higher content of SFA in *Chlorella sorokiniana* KNUA114 and KNUA122 under mixotrophic conditions was observed compared to *Chlorella vulgaris* KNUA104. An enhancement of SFA and the saturation of unsaturated fatty acids enhanced the value of the bioresource as an industrially useful fatty acid, and an increase in the carbon chain suggests the possibility of producing high-value lipids (Chandra et al., 2015). These results suggest that the application of mixotrophic cultivation to the *Chlorella* strains enhances SFA content and saturates the polyunsaturated fatty acids. Therefore, mixotrophic cultivation might be useful to improve and develop the *Chlorella* strains as a bioresource for producing high-value lipids. Because *Chlorella sorokiniana* KNUA114 and KNUA122 strains produce higher SFA content than *Chlorella vulgaris* KNUA104, the *Chlorella sorokininana* strains were therefore considered to be the most suitable strain for industrial applications.

**TABLE 2:** Fatty acid composition of *Chlorella* according to the cultivation conditions used, as useful biological resources.

Fatty acid	KNUA104		KNUA114		KNUA122	
	Photoautotrophic	Mixotrophic	Photoautotrophic	Mixotrophic	Photoautotrophic	Mixotrophic
C14:0	0.16 ± 0.05	0.25 ± 0.04	0.20 ± 0.03	0.37 ± 0.03	0.25 ± 0.05	0.46 ± 0.04
C15:0	0.06 ± 0.01	0.13 ± 0.01	0.06 ± 0.01	0.07 ± 0.01	0.03 ± 0.01	0.07 ± 0.01
C16:0	22.63 ± 0.36	25.49 ± 0.64	26.28 ± 0.98	33.75 ± 0.85	27.50 ± 0.02	30.86 ± 0.03
C16:1	1.80 ± 0.08	1.68 ± 0.77	1.40 ± 0.09	2.05 ± 0.08	1.33 ± 0.02	2.95 ± 0.09
C16:2	6.15 ± 0.15	4.53 ± 0.21	11.61 ± 0.22	2.04 ± 0.00	10.57 ± 0.05	3.23 ± 0.04
C16:3 ω3	9.28 ± 0.05	3.52 ± 0.04	14.88 ± 0.05	5.78 ± 0.06	14.58 ± 0.01	4.88 ± 0.04
C18:0	0.48 ± 0.00	0.00 ± 0.00	0.71 ± 0.01	0.00 ± 0.00	0.69 ± 0.00	0.00 ± 0.00
C18:1 ω9	11.03 ± 0.95	34.04 ± 3.30	3.34 ± 0.39	21.16 ± 0.49	2.93 ± 0.60	22.25 ± 0.49
C18:2 ω6	21.51 ± 0.91	16.86 ± 0.79	20.35 ± 0.29	20.73 ± 0.35	20.62 ± 0.40	21.63 ± 0.35
C18:3 ω3	26.90 ± 0.81	9.61 ± 0.94	21.17 ± 0.96	11.23 ± 0.26	21.50 ± 0.30	11.69 ± 0.26
C19:0	0.00 ± 0.00	3.89 ± 0.62	0.00 ± 0.00	2.82 ± 0.09	0.00 ± 0.00	1.98 ± 0.09
SFA^a^	23.33 ± 0.42	29.76 ± 1.31	27.25 ± 1.03	37.01 ± 0.98	28.47 ± 0.08	33.37 ± 0.17
UFA^b^	76.67 ± 3.37	70.24 ± 6.05	72.75 ± 2.00	62.99 ± 1.24	71.53 ± 1.38	66.63 ± 1.27
MUFA^c^	12.83 ± 1.03	35.72 ± 4.07	4.74 ± 0.48	23.21 ± 0.57	4.26 ± 0.62	25.20 ± 0.58
PUFA^d^	63.84 ± 2.34	34.52 ± 1.98	68.01 ± 1.52	41.78 ± 0.67	67.27 ± 0.76	41.43 ± 0.69

a Percentage of saturated fatty acids.

b Percentage of unsaturated fatty acids.

c Percentage of monounsaturated fatty acids.

d Percentage of polyunsaturated fatty acids.

### Cultivation Conditions Causing Compositional Changes and Resulting in Different Bioresource Productivity

The efficiencies of lipid, protein, and pigment production are summarized in Table 3. Efficiency for each component was calculated using the total yield, the additional yield resulting from the addition of glucose, and the additional yield per gram of consumed glucose. Lipid yield per gram of consumed glucose increases proportionally to the initial glucose concentration. However, lipid production reaches a threshold or decreases when in a specific initial glucose concentration for each strain (KNUA104: 15 g L^−1^; KNUA114: 20 g L^−1^; KNUA122: 20 g L^−1^). KNUA104 and KNUA114 show lipid yields per gram of consumed glucose of up to 0.20 g L^−1^, which was approximately 2-fold higher than the total lipid production achieved in the absence of glucose (KNUA104: 0.08 ± 0.01 g L^−1^; KNUA114: 0.10 ± 0.02 g L^−1^). Relative to photoautotrophic cultivation, the total protein yield increased in all cultures where glucose is added albeit varying per strain. Although KNUA104 and KNUA114 differ in the initial glucose concentration that induces the maximum total protein yield (KNUA104: mixotrophic condition, 10 g L^−1^; KNUA114: mixotrophic condition, 15 g L^−1^), both strains produced more protein per gram of consumed glucose at the lower glucose concentrations. Notably, the total protein yield of KNUA114 grown under either mixotrophic or heterotrophic conditions continued to increase as the initial glucose concentration increased. However, this trend was not observed in the values of protein yield per gram of consumed glucose; instead, these values remained relatively constant. The highest total pigment yield (0.36 ± 0.01 g L^−1^) was seen with the KNUA114 strain under mixotrophic conditions (glucose concentration: 20 g L^−1^). Both KNUA114 and KNUA122 had similar additional pigment yields, but total pigment yield was lower for KNUA122 than KNUA114 under the same conditions. For *Chlorella vulgaris* KNUA104, the maximum total pigment yield is seen under mixotrophic conditions (glucose concentration: 10 g L^−1^), and total pigment yield decreases at higher initial glucose concentrations. The results in Table 3 suggest that *Chlorella sorokiniana* KNUA114 most efficiently produces lipid, protein, and pigment. When KNUA114 was cultured at an initial glucose concentration of 15–20 g L^−1^, the efficient production of each useful substance was expected. Furthermore, when mixotrophic and heterotrophic conditions are compared, the yields of lipid, protein, and pigment were more frequently higher under mixotrophic conditions. Based on these results, *Chlorella sorokiniana* is a more appropriate strain than *Chlorella vulgaris* to cultivate in mixotrophic conditions.

**TABLE 3:** The compositional profile changes (lipid, protein, and pigment) after the addition of glucose to *Chlorella* under different initial glucose concentrations.

Strain	Initial glucose concentration (g L^−1^)	Total lipid yield (g L^−1^)	Additional lipid yield (g L^−1^)	Additional lipid yield per gram of consumed glucose (g g^−1^)	Total protein yield (g L^−1^)	Additional protein yield (g L^−1^)	Additional protein yield per gram of consumed glucose (g g^−1^)	Total pigment yield (g L^−1^)	Additional pigment yield (g L^−1^)	Additional pigment yield per gram of consumed glucose (g g^−1^)
*Chlorella vulgaris* KNUA104	0	0.08 ± 0.01			0.20 ± 0.02			0.02 ± 0.01		
	Mixotrophic									
	5	0.77 ± 0.06	0.69 ± 0.06	0.14 ± 0.01	0.64 ± 0.05	0.44 ± 0.05	0.09 ± 0.01	0.04 ± 0.01	0.02 ± 0.01	0.00 ± 0.00
	10	1.86 ± 0.06	1.78 ± 0.06	0.19 ± 0.01	0.97 ± 0.03	0.77 ± 0.03	0.08 ± 0.01	0.15 ± 0.01	0.13 ± 0.01	0.01 ± 0.00
	15	2.05 ± 0.11	1.97 ± 0.11	0.20 ± 0.01	0.60 ± 0.03	0.40 ± 0.03	0.04 ± 0.01	0.14 ± 0.01	0.12 ± 0.01	0.01 ± 0.00
	20	1.90 ± 0.06	1.82 ± 0.06	0.19 ± 0.01	0.82 ± 0.03	0.62 ± 0.03	0.07 ± 0.01	0.13 ± 0.01	0.11 ± 0.01	0.01 ± 0.00
	25	1.82 ± 0.11	1.74 ± 0.11	0.13 ± 0.01	0.88 ± 0.02	0.68 ± 0.02	0.05 ± 0.01	0.11 ± 0.01	0.09 ± 0.01	0.01 ± 0.00
	Heterotrophic									
	5	0.38 ± 0.02	0.30 ± 0.02	0.06 ± 0.01	0.53 ± 0.03	0.33 ± 0.03	0.07 ± 0.01	0.07 ± 0.01	0.05 ± 0.01	0.01 ± 0.00
	10	0.54 ± 0.14	0.46 ± 0.14	0.05 ± 0.02	0.64 ± 0.16	0.44 ± 0.16	0.05 ± 0.02	0.07 ± 0.01	0.05 ± 0.01	0.01 ± 0.00
	15	0.61 ± 0.01	0.53 ± 0.01	0.06 ± 0.01	0.37 ± 0.01	0.17 ± 0.01	0.02 ± 0.01	0.10 ± 0.01	0.08 ± 0.01	0.01 ± 0.00
	20	0.54 ± 0.08	0.46 ± 0.08	0.04 ± 0.01	0.46 ± 0.07	0.26 ± 0.07	0.02 ± 0.01	0.07 ± 0.01	0.05 ± 0.01	0.00 ± 0.00
	25	0.66 ± 0.04	0.58 ± 0.04	0.05 ± 0.01	0.45 ± 0.02	0.25 ± 0.02	0.02 ± 0.01	0.06 ± 0.01	0.04 ± 0.01	0.00 ± 0.00
*Chlorella sorokiniana* KNUA114	0	0.10 ± 0.02			0.16 ± 0.03			0.03 ± 0.01		
	Mixotrophic									
	5	0.51 ± 0.01	0.41 ± 0.01	0.08 ± 0.01	0.75 ± 0.01	0.59 ± 0.01	0.12 ± 0.01	0.10 ± 0.01	0.07 ± 0.01	0.01 ± 0.00
	10	1.32 ± 0.06	1.22 ± 0.06	0.12 ± 0.01	1.14 ± 0.05	0.98 ± 0.05	0.10 ± 0.01	0.14 ± 0.01	0.11 ± 0.01	0.01 ± 0.00
	15	2.22 ± 0.09	2.12 ± 0.09	0.16 ± 0.01	1.08 ± 0.04	0.92 ± 0.04	0.07 ± 0.01	0.27 ± 0.01	0.24 ± 0.01	0.02 ± 0.00
	20	2.11 ± 0.33	2.01 ± 0.33	0.20 ± 0.03	1.34 ± 0.21	1.18 ± 0.21	0.12 ± 0.02	0.36 ± 0.01	0.33 ± 0.01	0.03 ± 0.00
	25	1.96 ± 0.36	1.86 ± 0.36	0.14 ± 0.03	1.44 ± 0.27	1.28 ± 0.27	0.10 ± 0.02	0.26 ± 0.01	0.23 ± 0.01	0.02 ± 0.00
	Heterotrophic									
	5	0.33 ± 0.03	0.23 ± 0.03	0.05 ± 0.01	0.51 ± 0.05	0.35 ± 0.05	0.07 ± 0.01	0.09 ± 0.01	0.06 ± 0.01	0.01 ± 0.00
	10	1.15 ± 0.02	1.05 ± 0.02	0.11 ± 0.01	1.03 ± 0.12	0.87 ± 0.12	0.09 ± 0.01	0.15 ± 0.01	0.12 ± 0.01	0.01 ± 0.00
	15	2.08 ± 0.23	1.98 ± 0.23	0.15 ± 0.02	1.14 ± 0.13	0.98 ± 0.13	0.07 ± 0.01	0.29 ± 0.01	0.26 ± 0.01	0.02 ± 0.00
	20	1.62 ± 0.10	1.52 ± 0.10	0.11 ± 0.01	1.17 ± 0.07	1.01 ± 0.07	0.07 ± 0.01	0.24 ± 0.01	0.21 ± 0.01	0.02 ± 0.00
	25	1.77 ± 0.19	1.67 ± 0.19	0.13 ± 0.01	1.26 ± 0.14	1.10 ± 0.14	0.08 ± 0.01	0.20 ± 0.01	0.17 ± 0.01	0.01 ± 0.00
*Chlorella sorokiniana* KNUA122	0	0.09 ± 0.01			0.14 ± 0.02			0.02 ± 0.01		
	Mixotrophic									
	5	0.38 ± 0.04	0.29 ± 0.04	0.06 ± 0.01	0.70 ± 0.09	0.56 ± 0.09	0.14 ± 0.02	0.11 ± 0.01	0.09 ± 0.01	0.02 ± 0.00
	10	1.10 ± 0.08	1.01 ± 0.08	0.10 ± 0.01	1.36 ± 0.18	1.22 ± 0.18	0.12 ± 0.02	0.17 ± 0.01	0.15 ± 0.01	0.02 ± 0.00
	15	1.84 ± 0.07	1.73 ± 0.07	0.13 ± 0.01	1.53 ± 0.06	1.39 ± 0.06	0.11 ± 0.01	0.19 ± 0.01	0.17 ± 0.01	0.01 ± 0.00
	20	1.88 ± 0.08	1.79 ± 0.08	0.14 ± 0.01	1.03 ± 0.04	0.89 ± 0.04	0.07 ± 0.01	0.23 ± 0.01	0.21 ± 0.01	0.02 ± 0.00
	25	1.81 ± 0.38	1.72 ± 0.38	0.14 ± 0.03	1.01 ± 0.21	0.87 ± 0.21	0.07 ± 0.02	0.16 ± 0.01	0.14 ± 0.01	0.01 ± 0.00
	Heterotrophic									
	5	0.22 ± 0.04	0.13 ± 0.04	0.03 ± 0.01	0.33 ± 0.06	0.19 ± 0.06	0.04 ± 0.01	0.06 ± 0.01	0.04 ± 0.01	0.01 ± 0.00
	10	1.00 ± 0.04	0.91 ± 0.04	0.09 ± 0.01	0.82 ± 0.06	0.68 ± 0.06	0.07 ± 0.01	0.15 ± 0.01	0.13 ± 0.01	0.01 ± 0.00
	15	1.42 ± 0.12	1.33 ± 0.12	0.11 ± 0.01	0.85 ± 0.07	0.71 ± 0.07	0.06 ± 0.01	0.17 ± 0.01	0.15 ± 0.01	0.01 ± 0.00
	20	1.32 ± 0.10	1.23 ± 0.10	0.12 ± 0.01	1.00 ± 0.07	0.86 ± 0.07	0.08 ± 0.01	0.23 ± 0.01	0.21 ± 0.01	0.02 ± 0.00
	25	0.70 ± 0.36	0.61 ± 0.36	0.05 ± 0.03	0.59 ± 0.31	0.45 ± 0.31	0.03 ± 0.03	0.07 ± 0.01	0.05 ± 0.01	0.00 ± 0.00

### Efficacy of Organic Carbon Source in the Production of Microalgae Biomass

Biomass production by *Chlorella* was enhanced as a result of applying mixotrophic and heterotrophic conditions (Table 1). Subsequently, this increased biomass production enhanced the production of lipids, proteins, and pigments (Figures 4A–I and Table 3). However, there are clear limitations to enhancing biomass productivity under mixotrophic and heterotrophic conditions. Although the productivity of biomass is enhanced by providing an organic carbon source (glucose), it is necessary to consider the increase in production cost (Patel et al., 2020). According to our results, the amount of biomass produced per gram of glucose consumed was similar to, or lower than, the amount of biomass produced under photoautotrophic conditions (Table 1). Considering the increased production cost, it would be inefficient to produce biomass by applying mixotrophic and heterotrophic conditions. However, when evaluating the production efficiency of biomass, considerations for both quality and quantity must be evaluated (Pereira et al., 2021). Although the production of protein and pigment was not high, and the amount of improved protein and pigment per gram of glucose consumed was lower under mixotrophic and heterotrophic condition than under the photoautotrophic conditions (Table 3). There was also a significant improvement in lipid productivity (Tables 2, 3Moreover, under the mixotrophic and heterotrophic conditions, not only was the total lipid content improved (Figures 4A–C), the lipid productivity per gram of glucose consumed was superior to that under the photoautotrophic condition (Table 3). Furthermore, the biomass produced under the mixotrophic condition had a tendency to be enriched for SFA and the tendency for MUFA to be enriched in the composition of UFA (Table 2). Therefore, this suggests that the mixotrophic condition is a more suitable cultivation method than the photoautotrophic condition for producing biomass for SFA or MUFA (Mansouri and Nezhad, 2020). In previous studies, it has been demonstrated that the lipids of microalgae biomass can be enhanced through an organic carbon source (Mansouri and Nezhad, 2020; Yun et al., 2021). Microalgae cannot only utilize this external organic carbon as an energy source but also store it in the body in the form of lipids by utilizing it for lipid synthesis (Mansouri and Nezhad, 2020; Yun et al., 2021). This method is involved in the metabolic process that converts photosynthetic products, produced through their photosynthesis, into lipids (Patel et al., 2020; Mehta and Chakraborty, 2021) explaining why glucose, a product of basal photosynthesis, can enhance lipid content (Patel et al., 2020; Mehta and Chakraborty, 2021). This demonstrates the importance of understanding the underlying processes in explaining the quality change of microalgae biomass caused by mixotrophic and heterotrophic conditions. Furthermore, a deep understanding of the process by which lipids are synthesized provides a choice of available organic carbon sources (Yun et al., 2021) as microalgae cannot use all types of external carbon sources (Yun et al., 2021). Knowing the available organic carbon sources by understanding the lipid synthesis process enables an economically strategic approach in applying mixotrophic and heterotrophic conditions (Yun et al., 2021). By utilizing a cheaper organic carbon source, the high production cost, which is a disadvantage of mixotrophic and heterotrophic conditions, can be solved (Patel et al., 2020; Yun et al., 2021). Finally, although mixotrophic and heterotrophic cultivations have limitations related to production cost in producing microalgae biomass, we propose a cultivation method that can produce high-quality biomass in relation to lipids. Furthermore, we suggest that the high production cost can be overcome by using inexpensive raw materials by understanding the metabolic process involving external organic carbon sources.

## Conclusion

In this study, biomass productivity and yields of lipid, protein, and pigment were improved in *Chlorella* by applying mixotrophic and heterotrophic conditions. Initial glucose concentrations were varied, optimal concentrations were identified, and the contents of the produced bioresource were evaluated. The *Chlorella* strains tested had optimal growth occurred at an initial glucose concentration of approximately 15 g L^−1^, and the biomass productivity under mixotrophic conditions was higher than the heterotrophic conditions within strains at the same initial glucose concentrations. The KNUA104 had a remarkable enhancement in lipid content, and KNUA114 and KNUA122 showed remarkable enhancement in pigment content. In addition, the SFA content of *Chlorella sorokiniana* was higher than *Chlorella vulgaris*. Comparison of the yield of biomass and the components of the biomass produced, the productivity of *Chlorella sorokiniana* was superior. Therefore, *Chlorella sorokiniana* was the most suitable strain for producing biomass, preferably with an initial glucose concentration of 15–20 g L^−1^ under mixotrophic conditions. This study provides evidence of the enhancement of biomass productivity by the application of mixotrophic and heterotrophic conditions and suggests its use in producing industrially useful bioresources by the improvement of the quality of biomass.

## Data Availability

The raw data supporting the conclusions of this article will be made available by the authors, without undue reservation.
